# Rapid Changes of Body Weight after a Headstand: A Metrological Analysis

**DOI:** 10.1371/journal.pone.0124764

**Published:** 2015-05-19

**Authors:** Alejandro Acuña-Espinoza, Luis Fernando Aragón-Vargas

**Affiliations:** Human Movement Science Research Center, University of Costa Rica, San José, Costa Rica; West Virginia University School of Medicine, UNITED STATES

## Abstract

Despite recent rules from amateur wrestling sport-governing bodies intended to discourage extreme weight loss measures, wrestling culture still includes varied methods to make weight, including holding a headstand position immediately before stepping on the scale. The procedure, according to the notion, will reduce reported mass anywhere between 250 and 500g (weight between 2.45 and 4.89 N). The aim of this study was to compare any possible differences between the headstand procedure (HS) and a normal (CON) weight measure, using a metrological approach defined by the European Association of National Metrology Institutes. Seventeen adult men were weighed on a force plate before and after doing a headstand or standing normally for 30s. The order of treatment application was assigned randomly. Post-test weight was significantly larger than pre-test (mean±s.d.) (640.7±62.8 N and 640.3±62.7 N, respectively, p<0.0001) under both treatments. No treatment vs. time of test interaction was found. No significant difference was found between CON and HS weight (640.6±62.8 N and 640.9±62.9 N, respectively, p=0.3815). The metrological tests suggest that the statistical differences found are related to the force plate measuring errors in every pre-established time interval. The 45g (0.44 N) difference found between pretest and post-test lies within the uncertainty range identified for the equipment (±110 g or 1.08 N). In conclusion, a 30-second headstand has no significant effect on registered body weight. The small variations obtained were due to equipment-associated measuring errors. This experiment offers systematic empirical evidence to aid in the elimination of this unjustified practice among the wrestling community.

## Introduction

A belief among wrestlers and wrestling trainers stands that if a man remains in a headstand position for near 30 seconds, returning immediately afterwards to an upright position, his reported mass will decrease anywhere between 250 grams and 500 grams. This improves his chances to achieve a lower weight class. Some wrestlers apply this technique immediately before weighing for competition as their last effort to make weight. Several explanations for this claimed decrement have been put forward but, to our knowledge, a formal scientific test of this issue has not been published. As weight regulations become more restrictive and the National Collegiate Athletic Association (NCAA) encourages healthy nutrition and hydration practices, together with more comprehensive testing [1], wrestlers may resort to accessible methods to achieve a required weight.

Instead of authoritatively dismissing this practice as nonsense, a scientific approach should be used. The first step would be observation (the experience reported by many witnesses: wrestlers and coaches). A good look at conventional theory (what scientists would call sound physical foundations), i.e., what is already known, is necessary. It is also important to postulate possible alternative explanations to the alleged phenomenon and test them empirically.

The first reasonable explanations which would come to mind relate to equipment errors or chance. While neither one is in fact a systematic error (sometimes weight would be higher, sometimes lower), it is desirable to know the magnitude of the equipment associated error. A brief explanation is warranted: one would expect that an object with a known mass, e.g., 40.0 kg, would weigh exactly 40.0 kg every single time it is placed on a properly calibrated weight scale, but it does not. Objects which have been certified according to strict criteria are classified in specific weight classes and may be called calibration weights [2]. A calibration weight may be used to characterize the behavior of a measuring device. But our measurement problem involves human beings, which are a bit more complex than a static object.

Other somewhat reasonable explanations for the wrestler’s belief have been attributed to fluid movements within the body, and balance distortions. When measuring body weight, the human body is apparently in static equilibrium, but as in any live/dynamic body there exists a natural frequency [3] which could be understood as a slight “regular vibration” present in humans. This last phenomenon is possibly associated with and/or impacted by one or more factors (e.g. heart rate, anatomical postural changes, muscular fatigue, mass/fluids redistribution). This natural frequency may be registered by a scale or a force platform. A dramatic variation of regular posture such as remaining in a headstand position could alter this regular vibration pattern, possibly altering the previous natural frequency. Once returning to an upright position, these vibratory alterations could modify the pattern by which the vertical ground reaction force is being interpreted by a low frequency weight measuring device. This device registers weight values in order to obtain an averaged measure, the final reading reported. In short, the natural frequency of the human body might be disturbed enough to alter the weight readings.

In the end, *reported weight* could be different from the *true weight value* that should be actually reported by the equipment. This may not occur necessarily because of equipment error but because of changes in the person being measured. The aim of this study was to compare any possible differences between a normal (control) weight measure and that obtained after remaining in a headstand position for at least 30 seconds. We first evaluated the equipment used to measure body weight utilizing a strict metrological approach, thus supporting the quality of our reported results. This enables us to assess whether the differences—if any—were beyond equipment quantified error. To our knowledge, this is the first study that presents a systematic approach to this problem, using highly reliable equipment with its respective reported expanded uncertainty.

## Methods

### Experimental approach to the problem

The main variable under study was *body weight* (B.W.) in Newtons (N). There are several assessments related to the quality of a measure: precision, accuracy, indication error, expanded uncertainty and critical error. *Precision* [4] is the degree to which repeated measures on a same mass show the same result. *Accuracy* [4] estimates how close a weight measurement of a reference mass is to the true value of the mass. *Indication error* involves both precision and accuracy. It is the difference between a weight´s true value and that indicated by the equipment utilized to measure it at a specific time interval, expressed as:

$${\text{E}}_{\text{j}}={\text{ I}}_{\text{L}}-{\text{I}}_{0}\;-\text{ g}\cdot \sum {\text{m}}_{\text{ref}}$$ (1)

where I_L_ stands for an average weight indication in Newtons (N) at a determined load, I_0_ (N) stands for an average indication when the platform is completely unloaded and Σm_ref_ is the sum of the reference weights in kilograms (kg) used at a determined load, which is being multiplied by the gravitational force *in situ* g_corr_≈ 9.78 m/s^2^. *Expanded uncertainty* also involves precision and accuracy. It is an established range where the true value for a weight measurement can be effectively found. This range is defined for a specific time interval. Expressed as:

$$U=\text{ k}\cdot u_{c}\left(y\right)$$ (2)

where coverage factor k = 2 allows a more conservative approach by duplicating the combined uncertainty u_c_(y) which is composed of the square sum of several uncertainties. These uncertainties are calculated according to EURAMET/cg-18/v.02 [5], and the Guide for the Expression of the Uncertainty [6]. The c*ritical error* is the combined result of the indication error and the expanded uncertainty, expressed as:

Error(t, cp)= Ej (t, cp) ± U(t,cp) (3)

where both E_*j*_ and U are exclusively time-interval dependent for our research condition (see section Measuring instrument performance under Results). Critical error, along with its mathematical components, makes it possible to report results knowing they are within a trusted range, relative to the measuring equipment used. In other words, it provides confidence that the weight being measured is a true weight, controlled by a range of an estimated error for that reported weight value. Consequently, when a participant’s weight is being measured, variations-if any- will be detected in a very precise and controlled way.

### Subjects

Seventeen sedentary adult men (22.5±3.4 years, 66.0±6.7 kg, 173.7±4.7 cm) volunteered to participate in this study. Statistical power calculations (β = 0.001) estimated that the n = 17 group, allows for the determination of a 250 g expected effect appropriately. All participants signed their written informed consent. The Science and Ethics Committee from the University of Costa Rica approved the research project. Regardless of habitual physical activity level, the compulsory requirement to participate in this study was their ability to remain in a headstand position for at least 30 seconds.

### Procedures

#### Instrumentation

All vertical ground reaction force (GRF) determinations were performed through direct data acquisition using a force platform (Bertec, OH, USA; Model: 6090–15) at a sampling rate of 1000 Hz for a 60 s period for each repetition. This instrument is much more reliable than conventional scales utilized at most regulated sport competitions. The platform’s critical errors were determined for each of 16 test time intervals considered for the total time of each weight measurement repetition: 0–1 s, 1–2 s, 2–3 s, 3–4 s, 4–5 s, 5–10 s, 10–15 s, 15–20 s, 20–25 s, 25–30 s, 30–35 s, 35–40 s, 40–45 s, 45–50 s, 50–55 s and 55–60 s. The expression of the measured weights resulted:

Weight (N)= Reported weight (N)+ Error (4)

applying Eq 3 becomes,

Weight (N)= Reported weight (N)+Ej (t, cp) ± U(t,cp) (5)

As explained before, the U is a derivate result of the u_c_ (y). This last value was determined applying a series of tests that comprised the device calibration procedure.

#### Testing protocol

Upon arrival for testing, participants read, discussed and signed the experimental protocol consent form. Participants were randomly assigned to one of two groups, which performed the tests in different sequences: one group was measured before and immediately after standing normally for 30 seconds; then they were measured before and immediately after 30 seconds in a headstand position. Individuals in the other group were measured before and immediately after 30 seconds in a headstand position first and then before and immediately after standing normally for 30 seconds. Each participant was weighed a total of 4 times: before and after the headstand treatment, and before and after the control. Each time the participants were measured, they stepped on the force platform and stood still in the fundamental anatomical position. They placed their feet over the platform calibrated areas, to register B.W. for a total of 60 s. Raw data were exported to a Microsoft EXCEL spreadsheet to determine average values for each of the 16 predefined time intervals mentioned above. This information was then analyzed through inferential statistics.

### Statistical Analysis

B.W. was analyzed using repeated measures 2X2X16, 3-way ANOVA design (condition by time interval by treatment). Statistical significance was chosen at p<0.05.

## Results

### Measuring instrument performance

Five calibration points (0, 40, 80, 120, and 140 kg) were chosen to test the force platform. These calibration points (cp, Eq 5) were used during both instrument calibration and participants testing. All the participants were found to be between the 40 kg cp (≈391 N) and the 80 kg cp (≈782 N), specifically between 581 and 713 N. Between these cps, the higher indication errors and *U* found for all the test time intervals during the calibration procedure belonged to the 80 kg cp. This allowed the definition of all the *critical errors* for each time interval on that same cp, becoming exclusively time dependent. Therefore, Eq 5 can be expressed as:

Weight= Reported weight+Ej (t) ± U(t) (6)

**Fig 1:**
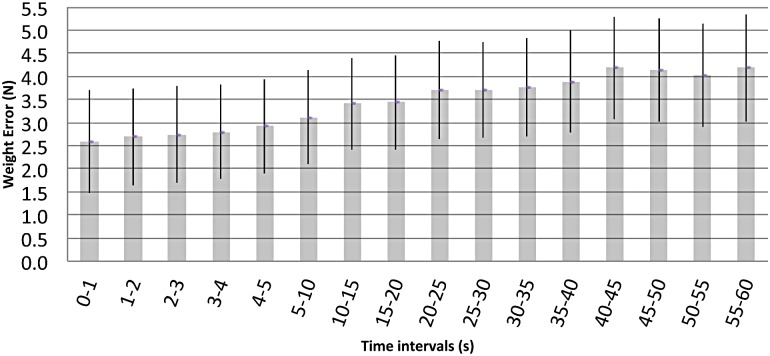
Error (N) associated to 80 kg cp by time intervals. Bar heights represent indication error (Ej) and the vertical lines the expanded uncertainties (μexp).

The errors utilized to report the weight results for the different time intervals (Eq 3) are summarized in Fig 1. The first five seconds are reported one by one, because the body weight changes could be potentially more evident, if existent, than in the remaining 55 seconds. E_j_(t) was found to be unilateral, with a platform value higher than the reference mass value being evaluated. Also it increased over time. Meanwhile U(t) exhibited a bilateral behavior, thus defining a symmetric range (Fig 1). As an example, the error associated to the [0,1] s interval was found to be 2.6±1.1 N (Eq 3). An uncertainty of ±1.1 N is equivalent to a ±0,12 kg mass value. This indication error characterization makes it possible to control the true weight values over time, to provide even more confidence to the whole weight test process.

### Subject response to the treatment

**Fig 2:**
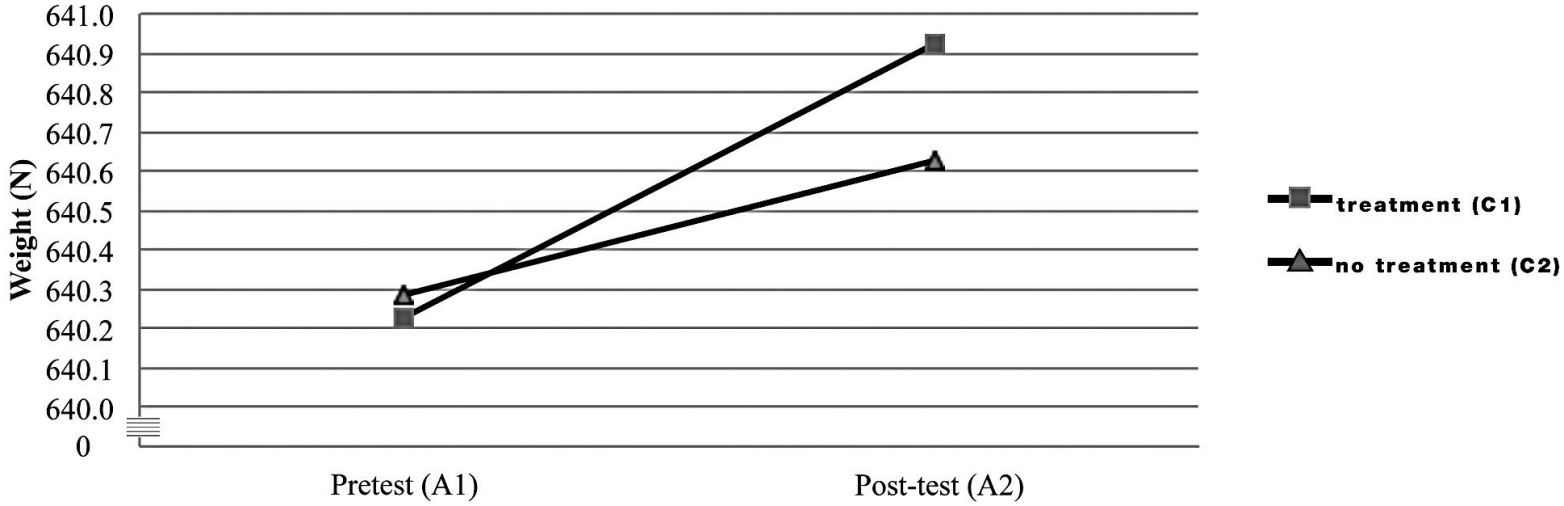
Interaction: order vs. treatment.

The results of the 3-way ANOVA are summarized in Table 1. There was no significant interaction between condition (A) and treatment (C) (p = 0.28), and no difference between treatment (C1) and no treatment (C2). No significant difference was found between normal weight and weight after a 30-second headstand (640.6±62.8 and 640.9±62.8N, mean±s.d. respectively, p = 0.3815) (Fig 2). However, post-test weight (A2) was significantly larger than pre-test (A1) (640.8±62.8 and 640.3±62.7N, respectively; F: 24.15*, p<0.0001).

**Table 1:** Three-way ANOVA: condition by time interval by treatment.

Source	F
**Pretest-Posttest (A)**	24.15*
**Time intervals (B)**	2.44*
**With/without headstand (C)**	0.81
A*S	
B*S	
C*S	
A*B	6.55*
A*C	1.25
B*C	0.43
A*B*S	
A*C*S	
B*C*S	
A*B*C	1.34
A*B*C*S	
**S**	
**T**	

There was a significant difference (*) between Prestest and Postest as well as among time intervals. Only the A*B interaction resulted significant (p<0.0001).

**Fig 3:**
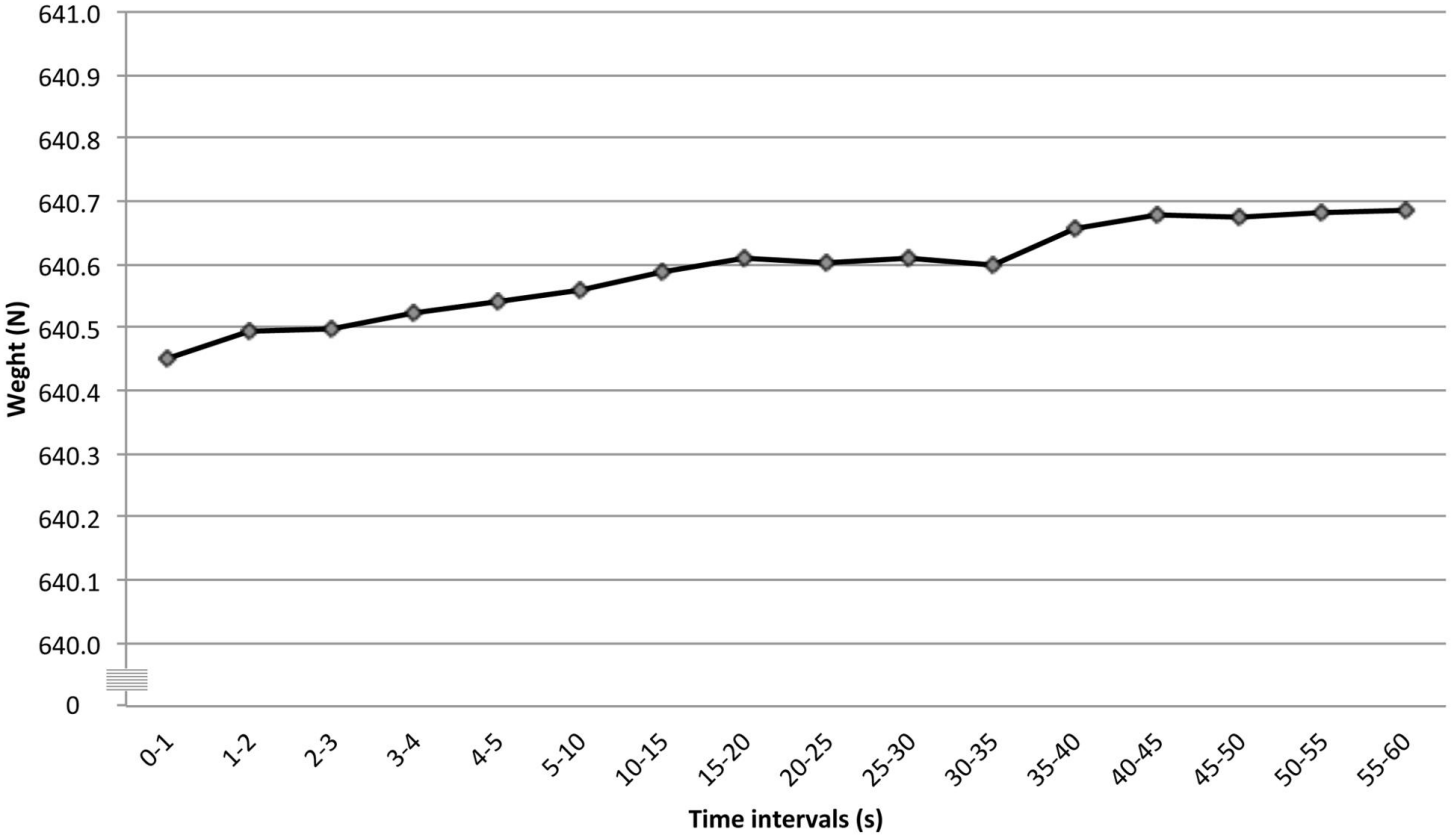
Averaged participants weight (N) measured at pre-established time intervals.

Results show that the Post-test average weight was greater than the Pretest by 0.44 N (approximately 0.045 kg). A significant difference was found among the test time intervals into which each test was divided (F: 2.43*, p = 0.0026; Fig 3). Additionally, the condition (A) vs. test time intervals (B) interaction was also significant (F: 6.55*, p<0.0001).

Overall, experimental results regarding the participant’s evaluation are consistent with the results exhibited during the platform calibration: participant’s weight measures increased over time at both pretest and post-test and during both testing sequences (Fig 3), as E_j_(t) did during calibration (Fig 2).

## Discussion

None of the results comparing the application to the non-application of treatment were found to be significant. In general, the experimental results suggest that the statistical differences found are directly related to the platform’s accuracy and are not due to the treatment. The main result from this study was that there is no effect of headstand (C) on weight variability. However, the analysis also suggests not only that the previously quantified platform’s E_j_(t) increment over time exists (F: 9.3, p = 0.0026) but also that the unloading time of the platform may lie beyond 30 seconds. This last issue is due to the statistical significance found between pre-test (A1) and post-test (A2) measures: the findings suggest that, after completely removing a load from the platform, it does not begin to register a complete unload after 30 s or more. The results regarding headstand are not surprising: B.W. should not be altered by a single postural change. It should be associated instead with other activities that cause significant mass changes in a relatively short period (a couple hours) such as exercise (which may lead to important fluid losses though sweating), or urinating.

We suggested that any potential weight changes after a headstand might be due to short-term changes in the natural frequency of the wrestler’s body. We did not detect any such changes (see Fig 3). However, we tested our participants under normal hydration (not assessed), but many wrestlers arrive at weigh-ins in a dehydrated state. To the extent that dehydration might alter changes in the natural frequency of the body due to sudden body position changes, this would be a limitation of the present study.

The experimental design based upon the random assignment of the treatment order and also utilizing the same participants for both experiment and control conditions, provides a pertinent approach to the research problem, effectively contributing to variance control. In human locomotion, the highest voluntary frequency is less than 10 Hz [7] consequently a low-frequency of 20 Hz according to the Sample Theorem [8] would be more than enough to minimize movement artifacts. A useful biomechanical sample frequency of 1000 Hz was utilized, thus providing a very accurate, time dependent weight measurement that would minimize the risk of missing peak values [7]. In contrast, digital scales and other potential weight measurement equipment provide limited measuring frequencies: their readings may lead to overestimating or underestimating the actual weight changes—if existent.

It is important to point out here that guidelines from sport-governing organizations related to amateur wrestling include little to no detail regarding the precision of weighing equipment to be used and how to record weight measurements. FILA (the International Federation of Associated Wrestling Styles) requires "scales (without springs) with guaranteed precision" [9]. Weight categories are specified in whole kilograms. NCAA recommends that a digital scale be used for weigh-ins and that all scales used should be certified before the start of each season [10].

This research was conducted in such a way to allow high reproducibility, while being conservative on its approach by ensuring high compliance with metrological guidelines: it was possible to associate an error (cp. Eq 3) to each weight measurement in every pre-established time interval. This allows for a stronger analysis that gives precision and accuracy a critical role. The present study provides a strong mathematical approach, rather than being limited to a simple statistical analysis which poses the risk of omitting practical findings.

Considering both the results from the platform calibration and the human tests, the pretest weight with its reported associated error at the [0,1] s interval was found to be 640.3–2.6±1.1 N (cp. Eq 6), that is 637.7±1.1 N. Post-test weight at the same time interval was found to be higher: 640.8–2.6±1.1 N, or 638.2±1.1 N (Fig 2). The numerical difference found between the pretest and post-test results (0.045 kg) despite being statistically significant, was negligible in light of the 1.1 N (0.11 kg) uncertainty: the difference lies within the uncertainty range. If we assume a dramatic scenario, in which the measurements are compared at the lower and higher points of the uncertainty range at the [0,1] s time interval, subtracting 637.7–1.1 = 636.6 N from 638.2 N+1.1 N = 639.3 N will result in a 2.7 N (0.27 kg) difference.

The unidirectional behavior of the indication error (Fig 2) focuses the attention on the reported, time dependent, expanded uncertainties. Specifically, at the cp selected (80 kg), the maximum possible difference for our equipment would lie between the 55–60 s interval, being of approx 2.33 N (0.237 kg, Fig 1) when considering the expanded uncertainty upper and lower limits (subtracting the lower limit to the upper limit). Within the first 5 seconds, it would be reduced to a maximum of approximately 2.23 N (0.228 kg). Taking into account expanded uncertainties at each time interval (at the 80 kg cp), when calculating the highest possible differences at the same time intervals, for pretest and post-test weight results, these differences vary between 2.43 N (approx. 0.249 kg @ (1–2) s) and 3.04 N (approx. 0.311 kg @ (55–60) s). The average value for the maximum possible differences at each time interval is of 2.65 N (0.271 kg), a value over the minimum target difference of 0.250 kg. In other words, when considering the most critical scenario regarding the quantified measurement’s associated error, it is not possible to affirm the non-existence of mass differences below 0.271 kg within one minute. It can be affirmed that, if existent, average differences between the weight at pretest and post-test are of ≤0.271 kg and they cannot be attributed to the effect of remaining in a headstand position for approximately 30s. The numerical differences found among pretest and post-test results are much lower than the U calculated for them: in a practical sense, there is no difference between pretest and post-test results. In order to find them if existent, an even more accurate and exact measuring equipment should be used.

Finally, we dare to speculate that since we were able to quantify and to express a platform ‘drift’ and its significance (p = 0.0026), a difference in measured weight caused by the treatment (headstand), if present, should have been evident. The conducted research found no appreciable differences on a man’s weight after remaining in a headstand position for approximately 30 s. The belief that has been spread among the wrestling discipline community is not supported by the present study, finding no significant differences except for the 0.270 kg between pretest and post-test measurements, a difference that was independent from remaining in a headstand position.

## Practical Applications

This experimental study shows, with the support of a metrological approach, that the belief among wrestlers and wrestling trainers that reported mass will decrease if a man remains in a headstand position for about 30 seconds and returns immediately afterwards to an upright position—hence improving his chances to achieve a lower weight class—is false. This experiment offers systematic empirical evidence to aid in the elimination of this unjustified practice which, because of its popularity, warranted scientific testing. We dare to speculate that since we were able to quantify and to express a platform ‘drift’ and its significance (p = 0.0026), a difference in measured weight caused by the treatment (headstand), if present, should have been evident. No appreciable differences on a man’s weight after remaining in a headstand position for approximately 30 s were found. The belief is discarded by the present study, finding no significant differences except for the 0.270 kg between pretest and post-test measurements; this difference was independent from remaining in a headstand position. Therefore, the athlete’s effort to decrease his reported weight during the official weighing by remaining in a headstand position for about 30 seconds is futile.
